# Collaborative Filtering Algorithm-Based Destination Recommendation and Marketing Model for Tourism Scenic Spots

**DOI:** 10.1155/2022/7115627

**Published:** 2022-04-28

**Authors:** Kejun Lin, Shixin Yang, Sang-Gyun Na

**Affiliations:** College of Business Administration, Wonkwang University, 460 Iksandae-ro, Iksan, Jeonbuk, Republic of Korea

## Abstract

The information age of rapid development of tourism industry provides abundant travel information, but it also comes with the problem of information overload, which makes it difficult to meet the growing personalized needs of people. The traditional collaborative filtering recommendation algorithm (CFA) also suffers from the problem of data sparsity when the user population increases. Therefore, this study optimizes the CFA through the similarity factor and correlation factor and enhances the tourism sense of travel experience through the satisfaction balance strategy. The experimental results show that the improved CFA method has the highest average accuracy on the overall dataset and the best recommendation performance of the satisfaction balance strategy. Overall, the recommendation model in this study is useful for attraction selection of users and marketing optimization of travel companies.

## 1. Introduction

With the development of information technology and the Internet, network information is becoming an important source of information for the public to plan travel routes, and people are gradually entering the era of big data from the era of lack of information [1]. In the context of massive data, how to quickly find the information of the best value for users is of great significance, and various recommendation systems have emerged with the needs of users. The recommendation systems [2] involved in the existing literature mainly focus on a single user and have achieved good results in TV programs, music, movies, news, and so on. In the tourism industry, the recommendation system is still in the initial stage of development and needs to be continuously improved. Compared with recommendation systems such as movies and music, it is difficult to obtain the ratings of scenic spots in the tourism field, and the user's rating matrix is relatively sparse. In addition, the selection of travel routes usually needs to consider the preferences of multiple users, so a recommendation system that combines all users participating in travel is a research hotspot in the field of travel recommendation [3].

In the recommendation system based on collaborative filtering, the rating of a single user needs to be predicted first. However, when calculating the similarity between users or items, the traditional collaborative filtering algorithm does not consider the impact of the number of items jointly rated by users and the degree of correlation between ratings on the similarity. For example, two tourists with different interests may have fewer attractions at the same time. When the users have fewer common ratings, the traditional collaborative filtering algorithm cannot accurately measure the similarity of users [4]. Therefore, it is necessary to consider fusing the prediction results of a single user. Practice shows that the recommendation effect of mean value strategy and least pain strategy is better. The average strategy takes the user's average rating on the item as a comprehensive evaluation result, but does not consider the dissatisfaction of a few members. The least misery strategy selects the member's lowest rating on the item as the comprehensive evaluation result, which ignores the preference of the majority of members.

Recommendation system refers to defining a function *F* to calculate the probability that an item *i* ∈ *I* (I am the set of all items) is recommended to a certain user *u* ∈ *U* (*U* is the set of all users) [5]. Recommendation algorithms find the most interesting items for users by calculating probabilities. Algorithms are the core of recommendation systems, and using efficient and accurate recommendation algorithms is the key to achieving good recommendation results. According to the different recommendation principles, it can be divided into popularity-based, social network-based, demographic-based, content-based, collaborative filtering-based, model-based, and hybrid recommendation algorithms. Recommendation based on popularity is to recommend hot content to users first, which can cover most of the content needs. Recommendations based on social networks include neighborhood-based social recommendation and graph-based social recommendation algorithms [6]. Demographic-based is to use the basic information of users, including age, gender, and place of residence, to calculate the degree of correlation between users and then make recommendations to users. The content-based recommendation algorithm recommends content like the items that they were interested in to users based on the attributes of the item itself [7]. The collaborative filtering algorithm proposed by Goldberg et al. is based on the assumption that if users *X* and *Y* rate *t* items similarly or have similar behaviors; then, users will rate or behave similarly to other items. It collects the user's past behavior to obtain the user's explicit or implicit information about the product, obtains the relevance of the product or user, and then recommends based on the relevance. Hybrid recommendation algorithms can combine the advantages of multiple algorithms to improve the performance of recommendation systems [8]. Hybrid recommendation algorithms include weighted type, switching type, intersection type, feature combination type, waterfall type, feature incremental type, and metalevel type [9]. According to the characteristics of travel recommendation and user needs, the recommendation algorithm based on collaborative filtering can better meet the recommendation requirements without causing excessive calculation. Nilashi et al. [10] used expectation maximization to construct a multicriteria collaborative filtering recommendation system for travel and tourism. Mehrbakhsh et al. [11] developed a collaborative filtering recommendation system based on ontology and dimensionality reduction techniques. Li et al. [12] proposed a combined recommendation algorithm based on improved similarity and forgetting curve.

In the process of travel recommendation, this study firstly improves the user-based and item-based collaborative filtering algorithms. The improved CFA combines the similarity factor and the correlation factor, which can better solve the problem of data sparsity in travel recommendation. Secondly, on the basis of average strategy and least misery strategy, a new user preference fusion strategy—satisfaction balance strategy—is defined. The strategy comprehensively considers the user's local satisfaction and overall satisfaction. Finally, through the experimental analysis based on the relevant tourism dataset of the city of Chongqing, it is verified that the improved method in this study can effectively improve the quality of tourism recommendation.

## 2. Recommendation Method

Collaborative filtering algorithm (CFA) is one of the most commonly used recommendation algorithms in the field of e-commerce recommendation, which does not require users to actively provide information about their personal needs, but obtains their potential preferences based on existing rating records. This study is based on the key techniques of recommendation for CFA applications, including fusion methods and fusion strategies. The fusion method is divided into model fusion and recommendation fusion. Model fusion generates recommendation combinations based on user preference models. Recommendation fusion, on the contrary, requires fusion based on prediction scores of each user after obtaining the prediction scores based on traditional algorithms and can also fuse the list of recommended items. The commonly used fusion strategies in recommendation key techniques include mean strategy, least pain strategy, and happiest strategy. Masthoff et al. [13] evaluated through a series of experiments that multiplication strategy, mean strategy, least pain strategy, and pain avoidance mean strategy are better. Zhang et al. [14] analyzed through literature studies and found that the most used strategies are mean strategy, pain mean value avoidance strategy, and minimum pain strategy, but the applicability of these strategies varies for clusters with different characteristics.

### 2.1. Recent Neighborhood Recommendations

**Figure 1:**
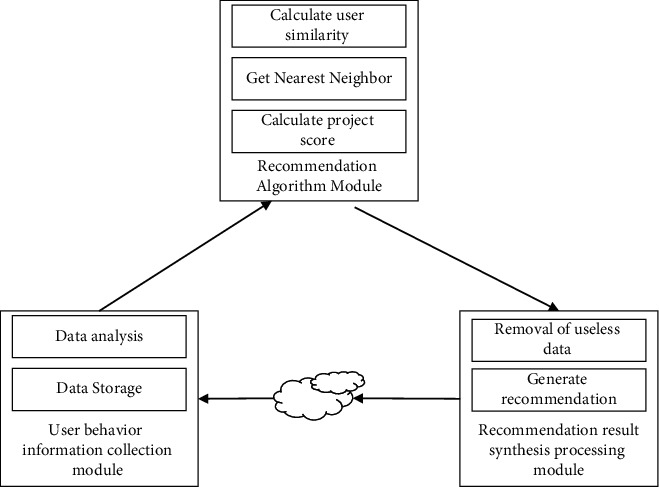
The framework of recommendation-based CFA.

CFA is often applied as a basic method in recommendation systems. The recommendation technique based on CFA includes four stages [15], such as similarity metrics, selecting neighbors, predicting ratings, and determining recommended items. Firstly, the similarity between every two users is calculated by the ratings of users in the rating matrix, then the ratings of current users for unknown items are predicted based on the K-nearest neighbor approach, and finally the recommendation list is generated by combining the preferences of all group members through a fusion strategy. The overall framework of the CFA-based recommendation technique is shown in Figure 1. Among them, CFA can be divided into user-based nearest neighbor recommendation and item-based nearest neighbor recommendation.

User-based nearest neighbor recommendation refers to the assumption that the current user will like the items liked by users with similar preferences. Currently, similarity calculation methods commonly used in practice include Cosine similarity and Pearson correlation coefficient. In this study, the main choice of similarity is defined as shown in the following equation:

(1) $$\begin{array}{c} c\text{sim}\left(p,q\right)=\frac{\sum_{i\in I_{pq}}R_{p,i}\cdot R_{q,i}}{\sqrt{\sum_{i\in I_{p}}R_{p,i}^{2}}\sqrt{\sum_{i\in I_{q}}R_{p,i}^{2}}}, \end{array}$$

where *c*sim(*p*, *q*) denotes the cosine similarity of users *P* and *Q*, *R*_*p*,*i*_ and *R*_*q*,*i*_ denote the ratings of user P and user *Q* for item *i*, respectively, *I*_*p*_ and *I*_*q*_ denote the set of items rated by user *P* and *Q*, and *I*_*pq*_ denotes the set of items jointly rated by user *P* and *Q*. By finding the set of users who have the similarity preferences to the current user *P*, as *KQQ*_*a*_, then the predicted ratings of user *P* for item *i* are as in equation (2), where $\bar{R_{p}}$ and $\bar{R_{q}}$ denote the average ratings of user *P* and *Q*, respectively:

(2) $$\begin{array}{c} G_{p,i}=\bar{R_{p}}+\frac{\sum_{q\in KQQ_{a}}c\text{sim}\left(p,q\right)\times \left(R_{q,i}-\bar{R_{q}}\right)}{\sum_{q\in KQQ_{a}}\left|C_{\text{sim}\left(p,q\right)}\right|}. \end{array}$$

The nearest collar recommendation based on the item uses the user's rating of the item to calculate the similarity, which is chosen in this study as shown in the following equation:

(3) $$\begin{array}{c} g\text{sim}\left(i,j\right)=\frac{\sum_{p\in R_{ij}}\left(R_{p,i}-\bar{R_{i}}\right)\left(R_{q,i}-\bar{R_{j}}\right)}{\sqrt{\sum_{p\in R_{ij}}{\left(R_{p,i}-\bar{R_{i}}\right)}^{2}}\sqrt{\sum_{p\in R_{ij}}{\left(R_{p,j}-\bar{R_{j}}\right)}^{2}}}, \end{array}$$

where *g*sim(*i*, *j*) denotes the Pearson similarity of item *i* and *j*, $\bar{R_{i}}$ and $\bar{R_{j}}$ denote the average scores of items *i* and *j*, and *R*_*ij*_ denotes the set of items that have scored both items *i* and *j*. By finding the set of items which have the similarity preferences to the current item *i*, as *KQQ*_*i*_, then the predicted ratings of user *Q* for item *i* are as in the following equation:

(4) $$\begin{array}{c} G_{p,i}=\bar{R_{i}}+\frac{\sum_{j\in KQQ_{i}}gsim\left(i,j\right)\times \left(R_{a,j}-\bar{R_{j}}\right)}{\sum_{j\in KQQ_{i}}\left|gsim\left(i,j\right)\right|}. \end{array}$$

### 2.2. Algorithm Improvement

Similarity is an important metric in CFA that determines how well a prediction is scored. In the travel field, the user's own combination of factors makes travel recommendations different from general e-commerce. For example, the frequency of users watching movies and online shopping in a year will be much greater than their frequency of travel. Therefore, the problem of data sparsity is more prominent in travel recommendation. The traditional similarity calculation method can give good results when the rating data are abundant, but in travel recommendation, the traditional recommendation method may ignore the influence of the sparsity of user rating data on the similarity calculation result when calculating the similarity between users or items.

Chongqing is one of the most popular tourist cities in China. This study takes the scoring matrix of famous scenic spots in the city by different users as an example to discuss this issue.

From the data in Table 1, the number of attractions rated jointly by users *A* and *B* is more than that of users *A* and *E*. Therefore, the similarity of users *A* and *B* should be higher. However, the cosine similarity between users *A* and *B* is calculated to be 0.724 and the similarity between users *A* and *E* is 0.871. Therefore, traditional similarity calculation methods cannot correctly account for the correlation between user ratings. To solve this problem, this study uses the relationship between the number of common user ratings of attractions and the total number of user ratings of attractions to adjust the similarity between users, that is, the similarity influence factor *s*_*i*_, and is defined as shown in the following equation:

(5) $$\begin{array}{c} s_{i}=2\sqrt{\frac{I_{pq}}{I_{p}\cup I_{q}}}-1, \end{array}$$

where *I*_*p*_ is the set of attractions rated by user *P* and *I*_*q*_ is the set of attractions rated by user *Q*. The larger the number of attractions jointly rated by users *P* and *Q* in the total number of rated attractions is, the larger the corresponding similarity influence factor and the larger the value of similarity may be; conversely, the smaller the value of similarity may be.

User *D* and User F have a cosine similarity of 0.835, indicating that they have similar preferences, while their Pearson and modified cosine similarity calculations are negative, indicating that they have opposite preferences. Both calculations deviate from the actual situation. As the size of users and rating matrices increases, situations like this can also affect the accuracy of recommendation results. To solve such problems, this study uses the correlation of user ratings to adjust the similarity between users; the closer the common rating vectors of users are, the larger the value of similarity may be and vice versa, the smaller the value of similarity may be. The correlation factor *s*_*i*_′ is defined as the following equation:

(6) $$\begin{array}{c} s_{i}^{\prime }=1-\sqrt{1-{\left[\frac{\sum^{}2R_{m,i}R_{n,i}}{\sum^{}\left(R_{m,i}^{2}+R_{n,i}^{2}\right)}\right]}^{2}}. \end{array}$$

The calculation of similarity is the most important step in collaborative filtering. The data sparsity problem faced by the travel domain makes it difficult for the original similarity methods to accurately measure the similarity among users. This is because in the case of sparse user rating data, traditional methods mainly consider the similarity between users' common ratings, but ignore the phenomenon that users are not necessarily similar on other items. Users' preferences can be considered similar only when they rate similarly on a relatively large number of items; moreover, traditional methods cannot accurately distinguish the similarity between some users with the same similarity but very different preferences.

The similarity influence factor *s*_*i*_ and correlation factor *s*_*i*_′ proposed in this study comprehensively consider the influence of common user rating items and rating correlation on the similarity measure, which can effectively alleviate the problem of inaccurate similarity calculation due to the data sparsity problem. Improved similarity *cs*im(*p*, *q*)_*i*_=*s*_*i*_[*θ* · *c*sim(*p*, *q*)+(1 − *θ*) · *s*_*i*_′], and *θ* is a parameter of [0, 1]. Through substituting *c*sim(*p*, *q*)_*i*_ into equation (2), we can yield the user's predicted score for the scenic spot.

### 2.3. Modified Preference Fusion Strategy

On the basis of individual users' prediction and scoring of items, the fusion strategy can fuse the user's preferences to obtain the overall evaluation value of each item and generate the final recommendation result according to the score. Since user preferences may vary, individual member preferences cannot represent overall preferences. How to obtain the common preferences of the overall users to alleviate the conflict is a problem that needs to be solved. Currently, the more commonly used preference fusion strategies include average strategy and least misery strategy. The average strategy selects the average of the user's rating on the item as the score of the item to be recommended. The calculation process is shown in the following equation:

(7) $$\begin{array}{c} R_{g,i}=\text{avg}\left(R_{a,i}:a\in g\right). \end{array}$$

The least misery strategy refers to taking the minimum rating of the item among users as the score of the item to be recommended, as shown in the following equation:

(8) $$\begin{array}{c} R_{g,i}=\text{min}\left(R_{a,i}:a\in g\right). \end{array}$$

The average strategy only considers the average preference degree of users participating in the rating and may ignore the dissatisfaction degree of a few users. The least misery strategy is based on the minimum rating of the user to evaluate the project, which may be the feeling of the majority of households. To this end, this study considers the shortcomings of the above two strategies and defines a satisfaction balance strategy, which is used to balance the relationship between the user's local satisfaction and overall satisfaction. The definition is shown in the following equation:

(9) $$\begin{array}{c} B_{g,i}=\text{avg}\left(R_{a,i}:a\in g\right)+\frac{1}{S}\;avg\left(R_{a,i}:a\in g\right)*\text{min}\left(R_{a,i}:a\in g\right), \end{array}$$

where *S* denotes the number of users who evaluates the items. Based on the above strategies, we list four users and their evaluations of the five attractions, as shown in Table 2. The table shows the attractions scores under the average strategy, the least misery strategy, and the satisfaction balance strategy. According to the average strategy, *S*_2_ and *S*_3_ are equivalent for the surveyed users, but for *S*_1_, the mean strategy does not consider the feelings of *U*_4_. According to the least pain strategy, *S*_1_ and *S*_2_ are equivalent to the surveyed users. Compared with *S*_3_, users are more interested in *S*_4_. The least misery strategy only considers the minimum satisfaction of members but ignores them, the preference of most people. Obviously, the result calculated according to the satisfaction balance strategy can better reflect the user's overall interest in the scenic spots. According to the revised fusion strategy, the recommended list can be obtained as *S*_4_, *S*_3_, *S*_2_, and *S*_1_.

## 3. Experimental Design

### 3.1. Experimental Data

There are no publicly available experimental datasets in the field of travel recommendation, and the data used in academic studies at home and abroad are mainly from travel websites or questionnaires. There are two problems in the data obtained by means of questionnaires in terms of data volume and subjective bias. In this study, we have crawled 2874 travel notes related to “Chongqing” from https://Qunar.com and use 49,318 scores as the experimental dataset, with a score ranging from 1 to 5 points.

In order to reduce the error, this study preprocesses the acquired data, and users with duplicate records for the same user, attractions with unclear ratings, and users with less than 3 rating records are removed. The final number of valid users is 4072, the total number of ratings is 25894, and the sparsity level is 0.965.

### 3.2. Accuracy Evaluation

Mean absolute error (MAE) and root-mean-square error (RMSE) are the two most common metrics for continuous variables. MAE is a linear score that represents the mean of the absolute error between the predicted value and the observed value. RMSE represents the sample standard deviation of the difference between the predicted and observed values (called residuals) and is used to indicate the degree of dispersion of the sample. In this study, the accuracy of individual prediction results is tested using MAE and RMSE, as shown in the following equation:

(10) $$\begin{array}{c} \text{MAE}\left(X,R\right)=\frac{1}{m}\overset{m}{\underset{i=1}{\sum }}\left|R_{i}-y_{i}\right|;\text{RMSE}\left(X,R\right)=\sqrt{\frac{1}{m}\overset{m}{\underset{i=1}{\sum }}{\left(R_{i}-y_{i}\right)}^{2}}, \end{array}$$

where *R* and *y* denote the predicted and true user ratings of the items in the test set, respectively, and *m* is the number of ratings.

There are many evaluation metrics commonly used in recommendation systems. The main idea of Discounted Cumulative Gain (DCG) is that a user's favorite item being ranked in front of the recommendation list will increase the user experience to a greater extent than being ranked at the back, as defined in the following equation:

(11) $$\begin{array}{c} \text{DCG}\left(b,L\right)=\overset{b}{\underset{i=1}{\sum }}r_{i}+\overset{L}{\underset{i=b+1}{\sum }}\frac{r_{i}}{{\text{log}}_{b}i}, \end{array}$$

where *r*_*i*_ indicates whether the product ranked *i* is preferred by the user, *r*_*i*_=1 indicates that the user likes the product, *b* is a free parameter, and *L* is the length of the recommendation list. Since DCGs are not directly comparable between users, we normalize them by dividing the original DCG by the ideal DCG to obtain the normalized discounted cumulative gain (NDCG). NDCG is a number between 0 and 1. The larger the value of NDCG, the more accurate the ranking of the items in the recommendation list and the higher the accuracy of the recommendation.

## 4. Experimental Results and Analysis

**Figure 2:**
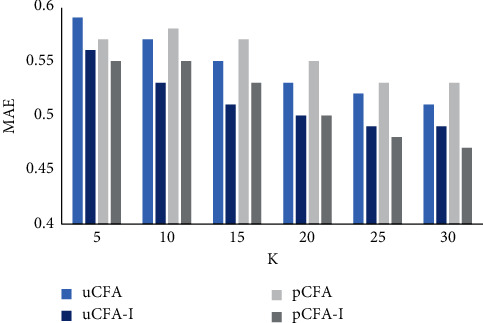
MAE comparison chart.

**Figure 3:**
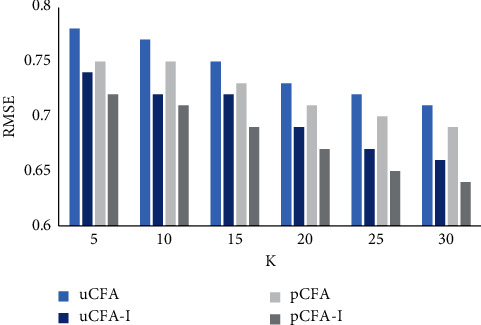
RMSE comparison chart.

We randomly select 70% of the rating records in the travel dataset as the training set and 30% as the test set and observe the performance of the recommendation algorithm when the number of neighbors *K* increases from 5 to 30 by 5 each time after several experiments, taking *θ*=0. From Figures 2 and 3, it can be obtained that, for the traditional user (uCFA)- and project (pCFA)-based methods, *s*_*i*_ and *s*_*i*_′ proposed in this study (uCFA-I; pCFA-I) are more effective in improving the CAF on MAE and RMSE. It shows that the improved method can provide better results in calculating the similarity between users or projects, which in turn improves the accuracy of prediction scores. The problem that traditional methods ignore the differences in users' interests for different attractions is reasonably solved.

**Figure 4:**
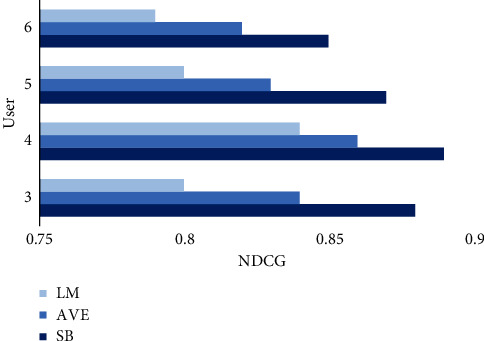
NDCG comparison chart.

Nowadays, people are more inclined to travel in groups, so it is important to fuse strategies for different users. Based on predictive scoring of users using a modified CFA, we compare the experimental results of the satisfaction balance strategy (SB) proposed in this study with the commonly used fusion strategies presented in Chapter 2.3 (AVE; LM) on a tour dataset. As can be seen from Figure 4, the NDCG values of the SB fusion strategy are the highest for different user sizes. AVE only considers the overall satisfaction of all members when recommending items to different users, but ignores the individuality of members' feelings; the LM strategy uses the opinions of a few members to decide the choice of the whole group, and the recommended items do not have a high probability of making the highest satisfaction of all members. And SB takes into account the relationship between overall and local user satisfaction, allowing the recommended projects to better reflect the preferences of the entire group.

Besides, regardless of the fusion strategy, the number of users at 4 makes NDCG take the highest peak. This also shows that, in the present social context, small groups of four are the most popular way to travel. On this basis, user satisfaction decreases as the number of people increases. This is also in line with the actual situation; as the more people there are, the greater the difference in interests is and the more difficult it is for the group to reach the peak of satisfaction with the same attraction.

Tourism market is very necessary for the development of cities. The model proposed in this study can help people to make decisions when traveling. Nowadays, there are various methods of travel recommendation, and only the method with outstanding accuracy and recommendation performance can stand out. In this study, we have proposed two improvement factors based on the common CFA, which can make the user get a better experience. Nowadays, travel companies should customize their marketing with full consideration of user preferences, so the data source for this study's improved CFA method is web travel data that incorporate user interests. As society develops and incomes increase, marketing of tourism should adapt to the trend of increasing personalized demand by creating a user demand base. Also, tourism companies can use the method in this study to obtain the user satisfaction of different attractions so that they can identify the attractions with more commercial value or business potential, improve their marketing and achieve greater economic benefits. For example, a group cruise or skiing program for four people can be developed at natural landscapes such as Fairy Mountain to make the tour small groups, reduce the probability of group members separating, and improve the overall satisfaction of the tour.

## 5. Conclusion

In response to the team-based nature of tourists, this study proposes a travel recommendation method that integrates users' personalized needs and maximizes team satisfaction based on the improved-CFA method. The method uses the massive rating information about tourist destinations on the web to build the basic framework of the travel recommendation method. The problem of sparsity of CFA data is solved by two factor corrections based on users and items. In the instance validation of https://Qunar.com, the improved CFA method in this study has significantly lower MAE and RMSE than the nonoptimized CFA method for different *K*. The satisfaction equalization strategy used in this study is also due to the conventional fusion strategy for different number of users. This shows the superiority of the method in this study and also greatly helps tourism companies to come up with better marketing strategies under complex market conditions. The recommendation algorithm in this study has room for improvement in accuracy, and future work will focus on the improvement of model accuracy and relevance of user preferences.

## Figures and Tables

**Table 1:** User rating matrix.

User	Hong Ya Dong	Dream Ordovician	Ciqikou ancient town	Wulong fairy mountain
*A*	5	3	3	1
*B*	3	3	0	4
*C*	4	5	4	0
*D*	3	5	0	2
*E*	5	0	4	0
*F*	5	3	0	0

**Table 2:** Different aggregation strategy examples.

Users	*S*_1_	*S*_2_	*S*_3_	*S*_4_
*U*_1_	5	4	4	5
*U*_2_	4	5	5	4
*U*_3_	3	2	4	5
*U*_4_	2	5	3	4
Average	3.5	4.00	4.00	4.50
Least misery	2.00	2.00	3.00	4.00
Satisfaction balance	5.25	6.00	6.00	6.75

Note: *S*_*n*_ denotes the different scenic spots; *U*_*n*_ denotes the different users.

## Data Availability

The labeled dataset used to support the findings of this study are available from the corresponding author upon request.
